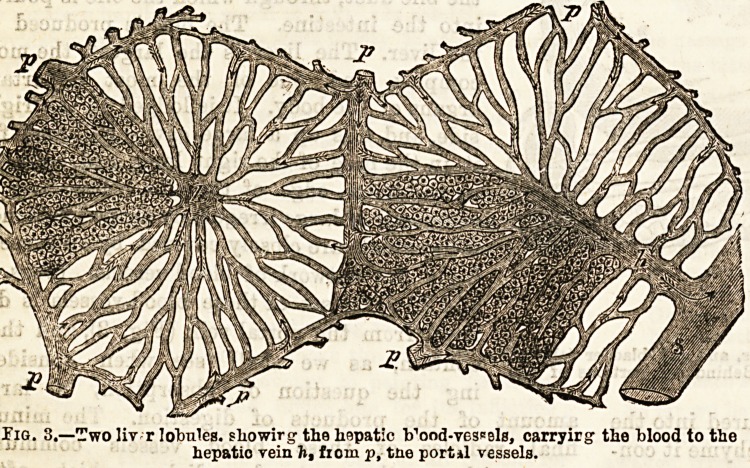# Diet in Disease

**Published:** 1892-12-10

**Authors:** Ernest Hart

**Affiliations:** Bachelier-ès-Sciences-es-Lettres (rèstreint), formerly Student of the Faculty of Medicine of Paris, and of the London School of Medicine for Women


### DIET IN DISEASE.

BY MRS. ERNEST HART, Bachelier-ès-Sciences-es-Lettres (rèstreint), formerly Student of the Faculty of Medicine of Paris, and of the London School of Medicine for Women.

### IX.—DIGESTION (*continued*).

**Digestion in the Duodenum.**—The duodenum is a strong muscular tube, about twelve inches in length (whence the name), which curves round the narrow head of the pancreas or sweetbread. At about the centre of the duodenum will be found the orifices of the tube or

FIG. I.—Relation of the stomach, duodenum, liver, and gall bladder (above), pancreas (below), and spleen (to right). Behind are portions of the aortic artery (right), and vena cava (left).

duct by which the pancreatic juice is poured into the duodenum, and becomes mixed with the chyme it contains. (See Fig. 1.)

**The Pancreas** is a glandular organ resembling the salivary glands in structure. It is concerned in secreting a fluid which has the very important parts to play in the digestive process of changing starch into sugar, and of emulsifying the fats. It has been already stated that it is necessary for insoluble starch to be converted into soluble sugar before it can pass through the walls of the blood vessels. The first step of this process commences in the mouth by the action of the saliva, but it is here incomplete, and it is stopped altogether as long as the food remains in the stomach. The substance called pancreatin, which forms 10 per cent. of the pancreatic juice, has the power almost instantaneously to change starch into sugar. There are various forms of sugar, and the form of sugar into which starch is changed by the action of the pancreatic juice in the duodenum is that known as glucose.

**Digestion of Fat.**—There remains one other large and important element of the food to be digested, namely, fat. Fat is not acted upon either by the saliva or by the gastric juices, but the instant it comes into contact with the pancreatic juice in the duodenum it undergoes what is called emulsification. Milk is the type of emulsified fat. If a drop of milk be examined under the microscope it will be found to consist of an immense number of very minute oil globules held in suspension in an albuminous fluid. In "setting the milk" these oil globules, being lighter than the rest of the fluid, rise and form the layer of cream. By the process of churning they are still further separated from the albuminous and other constituents of milk, and form a pure oily substance called butter. In order that the fat foods may be brought into a condition similar to that of milk, in which only they can be absorbed by the lacteals of the intestine, they must be emulsified or broken up into minute oil globules. This is effected by the action of the pancreatic juice, and fat once so emulsified remains in this condition. The digested food in the duodenum is called "chyle," and its reaction is alkaline.

**The Bile.**—About the level of the orifice of the pancreatic duct in the duodenum is found another small opening which is that of the bile duct, through which the bile is poured into the intestine. The bile is produced in the liver. The liver is the largest, the most complex, and one of the most important organs of the body. It is lodged in the right side, and fills up a large cavity hollowed for it in the base of the right lung, and bounded by the lower edge of the ribs. The liver is composed of large irregularly shaped flattened cells, which are closely covered by an exceedingly fine network of blood vessels. (Figs. 2 and 3.) One set of these blood vessels is derived from the portal vein (Fig. 3), and they contain, as we shall see when considering the question of absorption, a large amount of the products of digestion. The minute final radicles of these blood vessels communicate with another set of radicles, which after ramifying on the surface of the liver cells, collect into larger branches, and finally form the hepatic vein. The hepatic vein pours its contents into the vena cava or large blood vessel which conducts the blood to the right side of the heart (Figs. 1 and 3.) From this short description it will be seen that the products of digestion are brought into close relation with the liver

FIG. 2.—A liver lobule, showing the ramified blood vessels.

cells. The bile arises originally in the interstices between the liver cells, and by what are at first wall-less canals; they are minute ducts which contain an acrid greenish-brown substance known as the bile. These ducts gradually grow in size as they run together, and they finally pour their contents into a strong muscular tube by which they are conveyed to a hollow sack-like body called the gall bladder (Fig. 1). In the gall bladder the bile is stored for future use. At the moment that the food passes into the duodenum the bile is slowly poured out from the bile duct into the duodenum, and this discharge of bile continues during the whole process of digestion.

**The Uses of the Bile.**—It is allowed on all sides that the bile is a fluid of great importance in the digestive process, but what part it actually plays in this process has not yet been fully ascertained. Of its uses we are more persuaded when by some accident, such as the stoppage of the bile duct by a stone, or by an intentional operation diverting the flow of the bile outside the body, it ceases to be excreted into the duodenum. When no bile passes into the duodenum the patient or animal rapidly emaciates and may die of inanition. As far as we know at present the action of the bile is to emulsify the fats in the food, to precipitate, or throw down from the chyle all the partially digested and undigested particles, and also to exercise an antiseptic action on the food-mass in its long passage through the intestines. If the bile is deficient or is withdrawn entirely the food undergoes putrefactive changes in the intestine, with the production of flatus and of putrescent odours.

Fig. 3.—Two liv r lobules. showirg the hepatic b'ood-vessels, carryirg the blood to the hepatic vein *h*, from *p*, the portal vessels.

**Digestion in the Intestine.**—From the duodenum the food passes into the small intestine. The small intestine measures in the adult male seven and a-half yards long. For the whole of this length its internal surface or mucous membrane is closely set with small tubular glands called the crypts of Lieberkühn. These glands, which are present in countless millions, secrete and pour out into the intestine a watery alkaline fluid. The intestinal juice has, though in a much smaller degree, the same properties as the more active juices of the stomach and pancreas. Thus the process of digestion is continued in a lesser degree throughout the whole tract of the intestinal canal, and the albumen which has escaped change into albumose in the stomach, and the starch which has not been converted into glucose by the action of the pancreatic juice in the duodenum, slowly undergo those necessary changes in the intestines.

The presence of food in the intestine acts as a stimulant to its muscular walls, and slow contraction of the involuntary muscular fibres of these walls takes place, by means of which a vermiform movement of the intestine is set up, which slowly passes the chyle on towards the large intestine.

**The Large Intestine and its Contents.**—The opening of the small intestine into the large is by a narrow slit called the ileo-cæcal valve. By the time that the chyle enters the large intestine its fluid particles and the large amount of intestinal juice thrown out by the crypts of Lieberkühn have been absorbed, and it has assumed a pasty consistence and acquired an offensive fæcal odour. The fæces contained in the large intestines consist of the indigestible remnants of the food, and various excretory materials thrown into the alimentary canal during the process of digestion. The undigested substances are the woody and fibrous parts of vegetable food. the elastic fibres and tissues or gristle and the insufficiently cooked parts of animal food. This collection of excrementitious materials being of no use whatever to the economy, it is gradually passed along the large intestine, and is thrown out of the body by the rectum.

## Figures and Tables

**Fig. I. f1:**
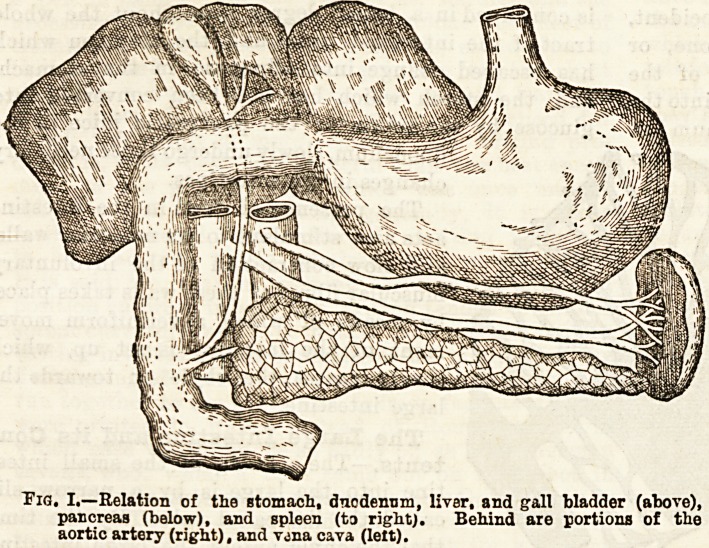


**Fig. 2. f2:**
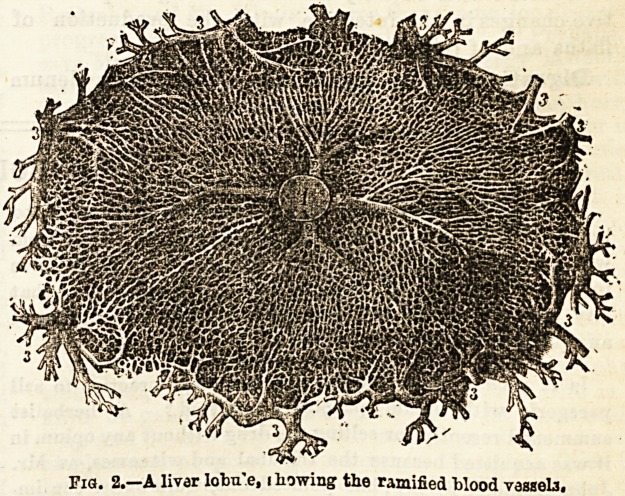


**Fig. 3. f3:**